# Trends in Immunization Completion and Disparities in the Context of Health Reforms: The case study of Tanzania

**DOI:** 10.1186/1472-6963-10-299

**Published:** 2010-10-30

**Authors:** Innocent A Semali

**Affiliations:** 1 Department of Epidemiology and Biostatistics, School of Public Health and Social Sciences, Muhimbili University of Health and Allied Sciences, P.O. Box 65015, Dar es Salaam, Tanzania

## Abstract

**Background:**

Of global concern is the decline in under five children mortality which has reversed in some countries in sub Saharan Africa (SSA) since the early 1990 s which could be due to disparities in access to preventive services including immunization. This paper is aimed at determining the trend in disparities in completion of immunization using Tanzania Demographic and Health Surveys (DHS).

**Methods:**

DHS studies randomly selected representative households from all regions in Tanzania since 1980 s, is repeated every five years in the same enumeration areas. The last three data sets (1990, 1996 and 2004) were downloaded and analyzed using STATA 9.0. The analysis included all children of between 12-23 months who would have completed all vaccinations required at 12 months.

**Results:**

Across the time periods 1990, 1996 to 2004/05 the percentage of children completing vaccination was similar (71.0% in 1990, 72.7% in 1996 and 72.3% in 2005). There was no disparity in completion of immunization with wealth strata in 1990 and 1996 (p > 0.05) but not 2004. In 2004/05 there was marked disparity as most poor experienced significant decline in immunization completion while the least poor had significant increase (p < 0.001). All three periods children from households whose head had low education were less likely to complete immunization (p < 0.01).

**Conclusion:**

Equity that existed in 1990 and more pronounced in 1996 regressed to inequity in 2005, thus though at national level immunization coverage did not change, but at sub-group there was significant disparity associated with the changing contexts and reforms. To address sub-group disparities in immunization it is recommended to adopt strategies focused at governance and health system to reach all population groups and most poor.

## Background

There is a global concern on the non-uniform decline of mortality rate in children under five a situation which has reversed in some countries, in sub Saharan Africa (SSA), since the early 1990 s [1]. Among 22 SSA countries only five were on track with the Millennium Development Goal 4 (MDG4), while rest recorded a reversal or a minimal decline [2]. The MDG4 call for reduction of child mortality by two thirds between 1990 and 2015, there was reversal in the decline of under five mortality most conspicuous in countries that also experienced decline in child immunization. Tanzania recorded stagnation in immunization coverage but with a decline in under five mortality [3]. Analysis of factors associated with increased infant mortality across countries has revealed markedly diverse correlates with immunization coverage suggesting non-uniform causes across countries, hence a need for country specific activities to raise immunization coverage[1]. Detailed analysis of data to understand specific correlates in each time period in each country is an important input towards development of strategies to achieve the MDG4 [4]. The success of the global and national strategies to raise immunization coverage could be enhanced by the understanding of factors affecting immunization uptake and those related to the stagnation of immunization coverage at household and community levels.

Child survival and immunization success is also influenced by the health system performance which had been experiencing low financing since 1980s; when Tanzania adopted Strategic Adjustment Program (SAP) and later, National Economic Survival Programmes (NESP) to foster economic development[5]. Following limited achievements, Economic Recovery Program (ERP) was established in 1986, and initiated political and economic liberalization processes. In 1990, it was followed by Public Sector Reform including decentralization by devolution, health sector reforms and restructuring of public administration to improve efficiency, effectiveness and donors support.

Donor relationship was enhanced through the adoption of Sector Investment Plan (SIP), Sector Wide Approach (SWAp), external debt relief and adoption of country owned National Strategy for Growth and Reduction of Poverty (NSGRP) with Kiswahili acronym MKUKUTA, to guide social and economic development. As a result funding modalities for vertical programmes like immunization were reformed and aligned to SWAp strategies, Basket Funding and integration of vertical programmes. In addition, the reforms and EPI integration took place in the context of high poverty which in 2000 was estimated at 40%. On the other hand, using basic needs approach to child poverty, it was estimated that over 80% of the children were in material poverty[6]. Families ranking low in the poverty scale would be more likely to be affected by the HIV and AIDS epidemic and less likely to complete immunization. In addition, such children would be less likely to access other health services, at higher risk of malnutrition and infectious diseases and hence posing a challenge in achieving the MDG4.

Global efforts to increase immunization such as the Global Immunization Vision and Strategy (GIVS) have urged countries to increase immunization coverage to 90% at the national level and at least 80% at the district level [7]. Other goals included urging countries to sustain immunization coverage, to ensure access to vaccines of assured quality and strengthening of health systems by 2015. In Tanzania, these goals have been translated at the national level by the 2007-2015 health strategic plans focused at raising national immunization coverage to 90% and child survival for the next ten years. The Expanded Program on Immunization (EPI) in Tanzania since 1977 adopted relevant interventions addressing the health system problems including the shortage of [8]. Streamlining immunization in Tanzania and the collaboration with other countries in the progress to achieve MDG4 also requires an understanding of the determinants contributing to the decline and stagnation of immunization coverage.

The DHS regularly collects systematic household data considered representative and of good quality to generate information to infer upon national level social development and health parameters [9]. DHS data collection has been done in the same Census Enumeration Areas and sample selection was designed to provide estimates for each regions or group of regions. Data obtained in 1990 coincides with baseline, a period just before reforms were implemented, while in 1996 it coincided with the start of implementing the reforms and 2004/05 which provided data collected ten years after the reforms.

This study therefore is set out to describe the influence of community and household factors on current trend in immunization of children in Tanzania and the observed stagnation of immunization coverage among children aged one year using available DHS. It is strongly suggested that analysis of the three data sets will enable researchers, policy makers and other stakeholders to understand the trend in determinants of immunization and suggest strategies that might contribute to higher and predictive immunization coverage in Tanzania.

## Methods

Tanzania is divided into 26 regions which are subdivided into districts with authority to plan and provide health services including immunization services. General objectives of the DHS were to determine child health, fertility rates and other health outcomes and their determinants in Tanzania. The DHS collects information using three questionnaires namely, the household questionnaire, female and male questionnaires. The female questionnaire among others, collects information on vaccination and health status of children less than five years of age. It was administered to 8138, 8,120 and 10,611 women in the reproductive age in 1990, 1996 and 2004/5, respectively. Access to the data was possible after submission of formal request to Measure DHS on http://www.measuredhs.com/myaccount/. The application was submitted through an account for a project titled **Equity and Immunization. **Itwas granted and data was downloaded for processing and analysis.

### Tanzania Health Care System and Reforms

The health system is organized into national, regional, district, ward and village levels. The immediate contact with the health system and also a source of preventive services are the health centres and dispensaries which together contribute 50% to the 4450 health facilities (public and private). Prior to the reforms, immunization of children operated vertically as expanded program on immunization (EPI) from the health centres, clinics and dispensaries.

The Tanzanian Government and Development Partners jointly developed and implemented health sector reforms beginning mid 1990 s. Reform activities were focused around 9 strategic areas within the health sector investment plan. Some of the implementation strategies included reorganization of health service management under LGR; strengthening drug supply system; and integration of numerous Vertical programs. Integration of EPI which was reported to have contributed to child survival was initiated late 1990 s to date.

### Completion of immunization

The DHS collected information on vaccination coverage for all children less than five years in a household in the three time periods. The number of children aged one year was involved. The results was that each time period were 1617 in 1990, 1 241 in 1996 and 1 613 in 2004/5. DHS collected data on immunization from road to health cards when available and mother's history and each vaccine received was recorded. A child was considered fully immunized if he/she had received *Bacillus Calmette-Guerin *(BCG) vaccine, three doses of Diphtheria, Tetanus and Pertusis toxoid (DPT) vaccine, four doses of oral poliovirus vaccine and measles vaccine.

DHS used multi-stage stratified design to obtain the study samples from all seven zones, urban and rural areas of Tanzania mainland and Zanzibar. Correction factors and weights have been calculated for each of the seven zones, urban, rural and Zanzibar. Thus the data and statistics which had been adjusted given the sampling design effect and hence the immunization coverage obtained in this analysis shall be representative of the national average.

### Household characteristics

At each interview wave, household rosters were compiled to appreciate the number of people in a household, their sex and age. Information on the number of years of education of the mother, father and head of the household was recorded. Information on the size of farm, quality of household, sources of water, ownership of toilet and assets was also gathered. As part of the objectives of the DHS, data on adult mortality indicators was also collected. This included orphan hood by type, occurrence of any adult death and presence of female headed household.

### Wealth index and quintile

Growing disparities in health outcomes are mainly explained by the continuing social, economic and political stratification of the population and the resulting inequalities[10]. In order to make progress towards equity several parameters have been proposed to measure health disparities and includes rate ratio, rate difference, low to high ratio between the strata. This study analysed immunization disparities between well-being strata stratified as wealth quintiles. Use of wealth quintiles to measure inequity was first introduced by Filmer and Pritchett[11-13], who used easily verifiable household data on durable asset (e.g. furniture, bicycle etc.) ownership, housing quality and infrastructure to measure wealth. Such parameter as a measure of wealth stratification is comparatively more permanent, valid and repeatable and its relationship with health outcomes has been consistently established in different set-ups[14,15].

The DHS questionnaire collected information on household possession of a number of consumer items ranging from a television set to a bicycle or car, as well as dwelling characteristics such as source of drinking water, type of sanitation facilities, and type of materials used in dwelling construction. Using principal component analysis each asset was assigned a weight (factor score) which were later standardized (DHS). Each household was then assigned a score for each asset, and the scores were summed for each household. Individuals were ranked according to the total score of the household in which they resided. The first factor with the highest loading value was adopted as the wealth index and quintiles of wealth were derived from it [16-18]. The wealth indices and wealth quintiles derived from the DHS datasets were used in this analysis. The sample was then divided into quintiles from most poor to fifth level as least poor. DHS data sets for 2004 and 1996 included wealth quintiles but for 1990 it was calculated using the same methodology and parameters.

### Analysis

Descriptive statistics included calculation of proportions of those who received each immunization antigen and those who had completed the immunization schedule. Using 1990 as baseline, changes in proportions of children aged one year completing their immunization schedule for 1996 and 2004/5 was calculated. Outcome variable (completion of immunization) was coded as binary variable (completion 1 and non-completion 0). Using STATA 9.0 software's Tabodds OR, command enabled studying the effect of multiple levels of wealth as exposure in addition to the dose response relationship by testing for homogeneity and trend[19]. Tabodds command yielded Crude Odds Ratio (OR) and adjusted OR (adjusted for other independent variables) with 95% Confidence Intervals (CI) for each independent variable controlling for other independent variables. In addition, score tests for trend and homogeneity were computed and statistical significance was set at p < 0.05.

## Results

This analysis included data on 1617, 1 241 and 1 613 children aged one year from the 1990, 1996 and 2004/5 DHS surveys respectively. Proportions of children completing immunizations were 71.0% in 1990, 72.7% in 1996 and 2004 it was 72.3%. Compared to 1990, proportion completing immunization increased slightly in 1996 (1.6%), but a bit lower (1.4%) in 2004 (Table 1). BCG and all DPTs compared to 1990 increased although slightly in 1996, but changes for all were negative by 2004. Other vaccines namely polio and measles followed similar trend increasing in 1996 and there after declining in 2004. For example measles completion was 81.1% in 1990 which increased by 2.4% to 83.5 in 1996 but declined by-0.6 to 80.5% in 2004 much lower than 1990.

**Table 1:** Changes in antigen coverage in 1996 and 2004/5 compared to 1990.

Antigen	Coverage year (%)			Difference (1990 as reference)	
	
	1990	1996	2004/5	1996	2004/5
BCG	95.9	96.8	92.2	0.9	-3.7
DPT1	95.4	95.6	93.8	0.2	-1.6
DPT2	91.2	92.2	90.6	1.0	-0.6
DPT3	80.5	86.7	86.7	6.2	6.2
					
Polio 1	94.7	96.1	94.2	1.4	-0.5
Polio2	89.5	92.7	90.8	3.2	1.3
Polio3	77.5	81.1	83.9	3.6	6.4
Measles	81.1	83.5	80.5	2.4	-0.6
Complete immunization	71.1	72.7	72.3	1.6	1.2

Table 2 shows changes in proportions of children aged one year who completed immunization schedule by selected household demographic characteristics during the year of demographic health survey. Proportion of children completing immunization in urban areas declined by 6.0% in 1996 and 4.6% by 2004, in the rural areas however, it increased by 2.5% to 70.0 in 1996 and, 2.0% in 2004. All age strata of heads of households compared to 1990 showed immunization gain in 1996, but in 2004 those aged 40 years and above recorded a decline. Similarly children from female headed households and those with lower education recorded a decline in 2004.

**Table 2:** Changes in proportion completing immunization by household demographic characteristic and year of survey.

Characteristic	Year			Difference	
	
	1990 N = 16	1996 (N = 1241)	2004 (N = 1613)	1996	2004
Urban	85.4	79.4	80.8	-6	-4.6

Rural	68.4	70.9	70.4	2.5	2.0

Age group					

< 30	72.8	74.4	73.7	1.6	0.9

30-39	70.2	73.1	75.4	2.9	5.2

40-49	72.2	72.3	70.9	0.1	-1.3

50+	69.8	71.1	67.3	1.3	-2.5

Female head of household	71.1	71.7	70.8	0.6	-0.3

Number of kids 1-2	73.9	73.8	74.2	-0.1	0.3

People in h/hold less than 5	70.1	75.2	75.4	5.1	5.3

Household head had less than 7 years of schooling	62.6	62.3	60.7	-0.3	-1.9

The changes in proportion of children aged one year completing immunization schedule by wealth quintile and year is shown in Table 3. Compared to 1990 immunization completion among the most poor increased by 10.6% to 75.0% which in turn declined by -3.8% to 60.6% in 2004; a trend which reversed among the least poor.

**Table 3:** Changes in proportion of children completing immunization by wealth quintile compared with 1990.

Quintile	1990	1996	2004/5	1996 Difference	2004 Difference
Most poor	64.4	75.0	60.6	10.6	-3.8

Very poor	70.5	71.3	72.1	0.8	1.6

Poor	60.7	69.9	71.8	9.2	11.1

Less poor	72	70.5	77.4	-1.5	5.4

Least poor	84.3	77.2	82.9	-12.1	-1.4

Table 4 depicts Tabodds tabulated crude and adjusted Odds Ratio for completion of immunization by household characteristics in 1990, 1996 and 2004/5. It revealed that being urban resident compared to rural a child was about three times likely to complete immunization (OR = 2.7, 95% CI 1.9-3.9), however in 1996 the difference was not significant, but in 2000 it was significant (OR = 1.4; 95%CI1.0-1.9). Similar trend was observed for those with more than two under five children compared to those with less. However, children of parents with less than primary school education were consistently less likely to complete immunization across all time periods in 1990 (OR = 0.6; 95%CI 0.5-0.7), while in 1996 it was (OR = 0.5; 95%CI: 0.3-0.7) and similarly in 2004 (OR = 0.4; 95%CI 0.3-0.5).

**Table 4:** Crude and adjusted odd ratios for completion of immunization by household characteristics and wealth by year.

Characteristic	1990		1996		2005	
	**Crude OR**	**Adjusted**	**Crude OR**	Adjusted*	**Crude**	Adjusted*

Residence						

Rural	1	1	1.0	1.0	1.0	1.0

Urban	2.7 (1.9-3.9)	2.3 (1.6-3.4)	1.5 (1.2-2.2)	1.3 (0.8-1.9)	1.8 (1.3-2.4)	1.4 (1.0-1.9)

Age group						

< 30	1	1	1.0	1.0	1.0	1.0

30-39	0.9 (0.6-1.2)	0.9 (0.6-1.4)	0.93 (0.7-1.3)	0.9 (0.6-1.4)	1.21 (0.8-1.5)	1.2 (0.8-1.7)

40-49	1.0 (0.7-1.4)	1.1 (0.7-1.8)	0.9 (0.6-1.3)	0.8 (0.5-1.3)	0.9 (0.6-1.2)	0.9 (0.6-1.4)

50+	0.9 (0.6-1.2)	1.2 (0.8-1.80	0.8 (0.4-1.2)	0.8 (0.5-1.4)	0.7 (0.53-1.01)	0.9 (0.6-1.3)

Head of household						

Male	1	1	1.0	1.0	1.0	1.0

Female	1.1 (0.8-1.5)	1.2 (0.8-1.7)	0.9 (0.6-1.3)	0.9 (0.6-1.4)	0.9 (0.6-1.2)	0.8 (0.6-1.2)

Number of children under five years						

Less than 2	1	1	1.0	1.0	1.0	1.0

More than 2	1.6 (1.3-2.1)	1.4 (1.1-1.9)	1.3 (0.9-1.7)	1.3 (0.9-1.8)	1.5 (1.2-2.0)	1.4 (1.0-1.8)

Number of people in the household						

< 4	1	1	1.0	1.0	1.0	1.0

≥3	0.8 (0.6-1.01)	1.0 (0.7-1.4)	1.2 (0.9-1.6)	1.1 (0.7-1.6)	1.3 (0.9-1.6)	1.1 (0.8-1.4)

Household head education (years)						

7 years or more in school	1	1	1.0	1.0	1.0	1.0

Not educated	0.5 (0.4-0.7)	0.6 (0.5-0.7)	0.5 (0.4-0.7)	0.5 (0.3-0.7)	0.4 (0.3-0.5)	0.4 (0.3-0.5)

Wealth quintiles						

Most poor	1	1	1.0	1.0	1.0	1.0

Very poor	1.3 (0.9-1.7)	1.3 (0.8-1.8)	0.83 (0.5-1.3)	0.96 (0.5-1.9)	1.7 (1.2-2.3)	1.5 (1.1-2.1)

Poor	0.8 (0.6-1.3)	0.6 (0.3-1.2)	0.8 (0.5-1.9)	0.8 (0.4-1.5)	1.7 (1.2-2.3)	1.4 (1.0-2.0)

Less poor	1.4 (1.0-1.9)	1.2 (0.7-2.0)	0.8 (0.5-1.2)	0.7 (0.3-1.9)	2.2 (1.6-3.1)	1.8 (1.3-2.6)

Least poor	2.9 (1.9-4.5)	1.7 (0.8-3.5)	1.1 (0.7-1.7)	1.0 (0.5-1.9)	3.2 (2.1-4.6)	1.9 (1.1-3.7)

P value for trend	< 0.001	0.06	0.47	0.02	< 0.001	< 0.001

*Adjusted for other independent variables.

Variation of completion of immunization among wealth quintiles in 1990 did not reveal a significant trend (p = 0.06). Similarly, the next period (1996) the trend was not significant (p = 0.06). On the contrary in 2004, the trend in completion of immunization increased significantly with improving wealth quintiles (p-value 0.001). Thus with increasing wealth, likelihood of completion of immunization increases significantly (p_trend _< 0.001). Unlike the two periods in 2004, children from the least poor households compared to the poorest were more likely to complete immunization (AOR = 1.9; 95%CI: 1.1-3.7).

## Discussion

This study determined the trend in immunization coverage, disparities and the influence of other community and household factors among children in Tanzania between 1990 and 2004. The analysis revealed the trend in completion of immunization was stagnant as it varied very slightly with time, it was 72.7 percent in 1990, 72 percent in 1996 and 72.3 percent in 2004. When it comes to antigen coverage for BCG, DPT1, Polio1 and Measles improved in 1996 and it declined markedly in 2004. On the other hand the rest of the antigens increased, thus compensating for the decline sustaining the coverage. Completion of immunization was also more likely in urban households in 1990 and in 2004, while during the three periods consistently low education of household head reduced the likelihood of child's immunization completion (OR = 0.6; 95% CI: 0.4-0.7) in 1990, in 1996 (OR = 0.5; 95%CI: 0.3-0.7) and (OR = 0.4; 95%CI: 0.3-0.5) in 2005. At the same time likelihood of immunization increased among households with more than two children who were less than five years old. There was no significant immunization disparity with wealth quintile in 1990 and 1996 but there was significant disparity in 2004.

Earlier DHS data analysis revealed disparities in the gains in immunizations (DPT3) as the richest were more likely to be immunized compared to the poorest[20]. This study found out that wealth was not significantly associated with completion of immunization in 1990 and later in 1996 suggests equity in completion of immunization in that period. The observed equity could have been a result of the EPI vertical strategies increasing access to immunization to all, with the goal to raise immunization coverage to 80% and above[21]. Among the vertical strategies of EPI was the use of multi-sectoral approach to boost immunization and soliciting high political commitment, health system commitment, community and household support. The outcome was increased awareness, high access to immunization services and high coverage. Consequently, there was no difference in completion of immunization between most poor and the least poor. However, in 2004 there was disparity in immunization as least poor and less poor compared to the rest had significant likelihood of immunization, thus suggesting the 1990 and 1996 strategies to increase access to immunization for the most disadvantaged had ceased to be effective.

Among households whose head had low education completion of immunization was significantly low. People with low education have limited access to information because they can't read hence they cannot comprehend health messages. In addition, given the low understanding, they would be more likely to have low incomes and hence unable to meet some costs related to getting a child immunized. Such population would benefit from vertical program strategies like outreach immunization services which are resource and human capital intensive. However, several studies done have associated non-completion of immunization with low education of the mothers or guardians[22-26]. Similarly, in this analysis low education of the head of the household was also associated with less likelihood of completing immunization which could reflect social status in such household affecting utilization of health care.

Existing evidence highly suggests countries that made good progress in primary health care and coverage of other interventions exhibited a high level of good governance[27,28]. Good governance included aligning and making sure all stakeholders including the community members are committed in achieving health and other development goals[29]. Thus such strategies were in place until 1996 which ensured that all those in the community had equal likelihood of accessing to immunization. Hence our finding that there was equity in completion of immunization based on wealth quintiles is explained by the EPI strategies that existed which made sure that all had equal access to immunization.

Reforms took place in the context of concerted donor support to vertical programmes and thus the equity in access in 1990. Donors support to vertical programmes was sustained until 1996 while planning and negotiating the reforms hence the continued equity in completion of immunization. After 1996, health reforms and decentralization were implemented with the aim of making better use of good practices and lessons learned from vertical programmes including EPI to benefit the rest of the health system[30,31]. Consequently reform strategies included integration of EPI activities to the rest of the health system, introduction of user fees and other changes in the health system but later observed to contribute to limiting access to health care[21]. As observed elsewhere limited access to health services was most pronounced among the poorest who were more likely to stop accessing health services compared to the well off people[32-34]. The reform processes and prevailing social-economic contexts would most likely explain the inequity which was observed in 2004 in this study.

There was increasing antigen coverage in 1990 although thereafter, 1996, there was a negative trend in the coverage of BCG, DPT and measles that coincided with reduction of the workforce, freezing of employment as well as diminishing budgetary allocation. Children and mothers obtained BCG and other immunizations at the level of dispensaries which was their first point of contact with the health system. The reduction in the likelihood of BCG immunization, other antigens immunization and consequently completion of immunization could also be explained by the shortage of health workers and other inputs observed in those health facilities[35]. The observations are further supported by a survey of 114 dispensaries in Tanzania which revealed that 40% of them did not have a qualified staff on the day of survey[22]. The facilities could not provide vaccines on the day of visit and more importantly BCG at birth thus raising the proportion of children not being vaccinated despite the fact that they had contacted the health system.

The observations presented above suggest that disparity in completion of immunization among children is derived from household, community and health system determinants. The current national policies suggests that it would ensure increased access to preventive services and equity though Community Based Health Care[36]. Community Based Health Care (CBHC) include strategies to reach those who are less likely to access health services including preventive services like immunization. Such national efforts are complemented by the global strategies including the global Immunization Vision (GIVS) whose objective is to reduce mortality rate by two-thirds by 2015 [37,38]. One of the lessons learnt therefore would be to ensure that there is a sustainable strong linkage between the global and national players, to take maximum benefits of such opportunities thus ensuring increased coverage to achieve the MDG 4.

The three periods analysed in this study could be taken to reflect an evolution of three phases namely: The Pre-1990 phase, characterised by robust vertical programmes like EPI, The 1996 phase characterised by stronger EPI programmes and transition to reformed systems. The third phase was 2004, phase characterised by implementation of reforms and integration of EPI to the rest of the health system. The phases mentioned implied major changes however; immunization variation was minimal, suggesting stagnation/sustenance. But there was significant differential decline in coverage among the most poor in 2005. That negative trend was offset by increasing coverage among the least poor. The result was undoing the equity achieved in completion in immunization in 1996, replacing it with gross inequity in 2004/5. There was recognizable stagnation masking inequity which evolved with time, reflecting changing contexts due to the reforms.

Reform effects at household could have been realized through changes that reduce the health system emphasis provided to EPI services, leading to service organization mode that needed more resources from a consumer to utilize it. Unfortunately, increased demand on consumer was easily borne by those who were well-off while those not well-off had to forego it leading to less likely of completing immunization.

Limitation to this study was the limited flexibility of the data sets as no possibility of adding extra data to further enrich the analysis raising some questions like; Given the household diversity there could be a need of obtaining data from different data sources in order to be able to make valid comparisons. DHS data sets were retrospective and could be out of date or the time intervals could not have been long enough for detecting trends. Such questions not withstanding, the DHS objectives and methodology included systematic collection of data on each immunization antigen during each period repeatedly in the same enumeration area. In addition the time interval of ten and fifteen years should be long enough for a trend to stabilize. The methodology was very rigorous minimizing data loses, at the same time data was collected from the same census enumeration areas each period thus making study population constant.

## Conclusions

An important lesson from this study is that strategies used 1990, 1996 and before achieved equity in access to immunization and therefore a need to sustain those strategies. Health sector reforms and change of strategies deprived those who were most poor access to immunization while increasing access among the least poor and thus creating inequity in access to immunization observed in 2004/5. Thus there is a need of adopting health promotion and provision strategies within the reform contexts in order to sustain equity in the completion immunization as was the case before 1996.

In conclusion, contrary to other periods, in 1996 there was no disparity in immunization coverage by any of the social characteristics except for education. Other periods revealed disparity in immunization coverage which was more marked with urban, education, household population and wealth indicators. Thus the analysis supports reports from other studies on health disparities with social characteristics, hence the need to have strategies to address the inequity. That not withstanding, equity prevailing in 1990 and which was more pronounced in 1996 could be a result of vertical nature of EPI which had strategies to reach all vulnerable including outreach services. With integration of EPI and other vertical programmes could have generated efficient implementation of the coverage strategies including outreach.

Recommendations arising from studies of health disparities also suggest that countries should consider research and developmental strategies to address all social determinants of health focused at all population groups[39]. It also calls for support for health system, governance, political as well as other sectors. It appeals for concerted efforts towards building more efficient, equitable and healthier societies. Sustenance and increased coverage of health interventions including EPI should have inclusive strategies embracing all actors with emphasis on community participation with plans to reach all population groups. Such interventions would require collaborative and continued monitoring of equity, documenting of good lessons emerging from implementation of vertical programmes packaged and disseminated regularly for governance and policy responses and interventions.

## Competing interests

The authors declare that they have no competing interests.

## Authors' contributions

Author participated in search and review of literature, applied for permission to use and analyse the data and wrote the manuscript.

## Pre-publication history

The pre-publication history for this paper can be accessed here:

http://www.biomedcentral.com/1472-6963/10/299/prepub
